# HLA-E: Presentation of a Broader Peptide Repertoire Impacts the Cellular Immune Response—Implications on HSCT Outcome

**DOI:** 10.1155/2015/346714

**Published:** 2015-08-12

**Authors:** Thomas Kraemer, Alexander A. Celik, Trevor Huyton, Heike Kunze-Schumacher, Rainer Blasczyk, Christina Bade-Döding

**Affiliations:** Institute for Transfusion Medicine, Hannover Medical School, 30625 Hannover, Germany

## Abstract

The HLA-E locus encodes a nonclassical class Ib molecule that serves many immune functions from inhibiting NK cells to activating CTLs. Structural analysis of HLA-E/NKG2A complexes visualized fine-tuning of protective immune responses through AA interactions between HLA-E, the bound peptide, and NKG2A/CD94. A loss of cellular protection through abrogation of the HLA-E/NKG2A engagement is dependent on the HLA-E bound peptide. The role of HLA-E in posttransplant outcomes is not well understood but might be attributed to its peptide repertoire. To investigate the self-peptide repertoire of HLA-E^*∗*^01:01 in the absence of protective HLA class I signal peptides, we utilized soluble HLA technology in class I negative LCL cells in order to characterize HLA-E^*∗*^01:01-bound ligands by mass-spectrometry. To understand the immunological impact of these analyzed ligands on NK cell reactivity, we performed cellular assays. Synthesized peptides were loaded onto recombinant T2 cells expressing HLA-E^*∗*^01:01 molecules and applied in cytotoxicity assays using the leukemia derived NK cell line (NKL) as effector. HLA-E in complex with the self-peptides demonstrated a shift towards cytotoxicity and a loss of cell protection. Our data highlights the fact that the HLA-E-peptidome is not as restricted as previously thought and support the suggestion of a posttransplant role for HLA-E.

## 1. Introduction

The classical class I human leukocyte antigens (HLA-A, HLA-B, and HLA-C) present allele-specific self or pathogenic peptides to CD8^+^ immune effector cells. The repertoires of peptides that can be presented by HLA molecules determine the diverse landscape of pHLA complexes in its entirety and thus the accessible interaction surface for a T-cell receptor (TCR). Even a single mismatch in the heavy chain (hc) of an HLA class I molecule can impact on the peptide binding profile and repertoire [1–8]. Since HLA class I molecules are highly polymorphic and most of their polymorphisms affect the peptide binding region (PBR) [6], it becomes obvious that systematic HLA matching is crucial in achieving successful allogenic hematopoietic stem cell transplantation (HSCT). For many hematologic malignancies HSCT is used as a curative treatment despite comprising posttransplantation complications related to the development of severe graft-versus-host-disease (GvHD), infection, and relapse. In order to achieve the best possible outcome when no HLA identical sibling donor is available, the matching of HLA-A, HLA-B, HLA-C, HLA-DRB1, and DQ between unrelated donors and recipients gives the best chance to maximize the success of an HSCT [9, 10].

In the course of minimization the risk of posttransplantation complications it has been suggested that the less polymorphic HLA-E could be responsible for posttransplant outcomes [11–13]. HLA-E belongs to the group of nonclassical HLA class I molecules and can be distinguished structurally from classical HLA class I molecules by two main features: (i) only 13 HLA-E alleles are known to date, although only two of them HLA-E^*∗*^01:01 and ^*∗*^01:03 have been shown to contribute to the molecules immune function [14]; (ii) the repertoire of known peptides that are presented through HLA-E molecules is very limited, suggesting that peptide-HLA-E (pHLA) complexes are relatively invariable ligands for the immune system. However, HLA-E molecules are known to be ligands for receptors of both the innate and adaptive immune system [15–21]. It becomes obvious that a larger repertoire of presented peptides than previously thought exists and provides immunogenic pHLA-E molecules that can stimulate innate and adaptive immune responses [22–25].

In the background of minor histocompatibility antigen- (mHag-) mediated GvHD in an HLA matched HSCT, a role of HLA-E has been addressed. It has been hypothesized that an out-competition of classical HLA molecules by HLA-E in the presentation of mHags could occur induced by its upregulation in the highly inflammatory environment posttransplantation [11]. This could in turn have a beneficial outcome in reducing the risk for GvHD that would otherwise be higher in HLA matched HSCT regarding mHAG incompatibility [26]. Another hypothesis is related to the role of alloreactive natural killer (NK) cells in T-cell depleted HSCT regarding the graft-versus-leukemia (GvL) effect of allografts in the recipient [27]. This effect is related to mismatched donor killer immunoglobulin-like receptors (KIR) and patient KIR ligands and is mediated by mature NK cells that emerge in the recipient several weeks following transplantation. In the early posttransplant period an NK cell population with the phenotype KIR^−^NKG2A^+^ is predominant. The affinity of NKG2A/CD94 and HLA-E is dependent on the bound peptide; hence the presentation of distinct peptides by HLA-E could influence the inhibition of these NK cells.

On the surface of healthy cells HLA-E is described to present a limited set of highly conserved hydrophobic peptides derived from classical HLA class I leader peptide sequences [28], and these complexes are ligands for the NKG2/CD94 receptor family of human NK cells [18, 20]. This receptor family comprises two receptors that solely recognize HLA-E, in particular the inhibitory NKG2A/CD94 and the stimulatory NKG2C/CD94 receptors [19]. Based on the interaction of the NKG2/CD94 receptors with HLA-E, NK cells can indirectly monitor the expression of HLA class I molecules within a cell. The presentation of HLA class I leader peptides through HLA-E is dependent on the health state of the cell [29]. In those cells undergoing cellular stress, HLA-E present fragments of heat shock proteins (e.g., HSP60) [30] and these pHLA-E complexes inhibit the binding to the NKG2A/CD94 receptor. The resulting abrogation of inhibitory signalling leads to subsequent NK cell mediated lysis that is triggered by activating signals from stimulatory receptors like NKG2D, NKp46, NKp44, or NKp30 [31–33]. HLA-E molecules have a protective function for healthy cells in innate immunity and complete the adaptive immune survey system that is mainly implemented through classical HLA molecules. Furthermore HLA-E molecules play an important part in viral interference, for example, human cytomegalovirus (HCMV) infections. During an HCMV infection classical HLA class I molecules are downregulated by viral immune evasion proteins, and the absence of classical HLA class I molecules makes the cells susceptible for NK cell mediated lysis [34]. To avoid this, the viruses evolved an effective method, making use of HLA-E molecules [35, 36]. The peptide VMAPRTLIL, derived from the UL40 protein of HCMV, mimics the signal peptide sequence present in most HLA-C allotypes; thus the HLA-E^VMAPRTLIL^ complex is able to circumvent NK cell mediated lysis by serving as a ligand for the NKG2A/CD94 receptor [37]. The VMAPRTLIL peptide has been reported to bind to HLA-E^*∗*^01:01 and as well to HLA-E^*∗*^01:03 [29]. The preservation of HLA-E by the HCMV derived peptide therefore allows the virus to escape from recognition by the immune system. However, an expansion of a NKG2C/CD94^+^ NK cell subset was reported in HCMV infected individuals, suggesting the presence of pHLA-E complexes prone to be ligands of the NKG2C/CD94 receptor [38, 39]. The expansion of this NKG2C/CD94^+^ NK cell subset after was reported to be indispensable from HLA-E expression on the infected cell as well as the presence of distinct cytokines as IL-12 and IL-15. However, the expansion of these NK cells was donor-dependent and it is not known if the expansion of NKG2C/CD94^+^ NK cells is restricted to the peptide that is presented by HLA-E.

Structural analysis of pHLA-E complexes in ligation with their receptors showed how the innate NKG2/CD94 receptors distinguish subtle differences in the amino acid (AA) sequences of the bound peptides [40]. The mechanism of how certain pHLA-E complexes activate or inhibit NK- or T-cell responses is however still not completely understood [16, 40].

A structural basis for the interaction between NKG2A/CD94 and HLA-E has been shown for HLA-E bound to the VMAPRTLFL peptide derived from HLA-G [41]. Indeed subtle changes in the AA composition of the bound peptide can influence the interaction between the NKG2A/CD94 or NKG2C/CD94 receptor complexes and HLA-E. The main contribution of the HLA-G derived peptide is mediated by the peptide residues p5-Arg and p6-Thr via hydrogen bonds to the CD94-Ser110 and CD94-Gln112, respectively. However, the NKG2A subunit contributes exclusively residue Pro-171 that interacts with the peptide position p5-Arg by van der Waals bond. AA variations at the peptides' p5 have been demonstrated to impact on receptor recognition [37].

So far, sequences and function of HLA-E restricted ligands from self or pathogenic origin are marginally identified. Utilizing soluble HLA technology [42] we determined the peptide features and variability of HLA-E^*∗*^01:01 bound peptides and their ability to impact on NK cell function.

## 2. Material and Methods

### 2.1. Cell Lines

HLA class I deficient B-LCL 721.221 and the TAP deficient T2 cell lines were maintained in RPMI 1640 (Life Technologies, Darmstadt, Germany) supplemented with 10% heat inactivated fetal calf serum (FCS), 2 mM L-glutamine, 100 U/mL penicillin, and 100 *μ*g/mL streptomycin (C-C-Prom Oberdorla, Germany). For production of lentivirus HEK 293T cells were cultured in DMEM (Life Technologies) supplemented with 10% heat inactivated FCS, 2 mM L-glutamine, 100 U/mL penicillin, 100 *μ*g/mL streptomycin, and 1 mg/mL Geneticin (Life Technologies). The human killer Ig-receptor (KIR)^−^ cytotoxic NK cell line (NKL) [43, 44] (kindly provided by Professor C. Falk, Hanover Medical School, Hanover, Germany) was cultured in RPMI supplemented with 15% heat inactivated FCS, IL-2 200 U/mL (PeproTech, Rocky Hill, NJ, USA) 2 mM L-glutamine, 1 mM sodium pyruvate, 100 U/mL penicillin, and 100 *μ*g/mL streptomycin. All cells were cultured at 37°C in a CO_2_ atmosphere of 5%. Polyclonal NK cells were obtained from PBMCs from healthy blood donors (Hanover Medical School).

### 2.2. Peptides and Antibodies

HLA-E restricted self-peptides, identified in this study, were synthesized and purchased from Thermo Fisher Scientific (Ulm, Germany) and dissolved in DMSO at a concentration of 100 mg/mL. For ELISA and affinity purification of soluble (s) HLA-E molecules the anti-HLA class I monoclonal antibody (mab) (clone W6/32) (AbD Serotec, Düsseldorf, Germany) or the mab anti-V5 (Life Technologies, Darmstadt, Germany) was used. The mab anti-*β*2m conjugated to horseradish peroxidase (HRP) (Dako, Hamburg, Germany) was used for detection in ELISA. The mab anti-HLA-E (clone 3D12) labeled with phycoerythrin (PE) (BioLegend, San Diego, USA) was used for flow cytometric analysis.

### 2.3. Cloning and Transduction of sHLA-E or mHLA-E Constructs

The cDNA fragments of mHLA-E^*∗*^01:01 (exons 1–7) and sHLA-E^*∗*^01:01 (exons 1–4) were amplified by PCR and cloned into the lentiviral vector pRRL.PPT.SFFV.mcs.pre as previously described [45]. For the production of lentiviral particles 6*∗*10^6^ HEK293T cells were transfected with 5 *μ*g of either pRRL/mHLA-E or pRRL/sHLA-E vector, psPAX2 (packaging plasmid), and pDM2G (envelope plasmid) by Lipofectamine 2000 (Life Technologies, Darmstadt, Germany). The B-lymphoblastic LCL 721.221 cells were then transduced with viral particles that contain the pRRL/sHLA-E and T2 cells with pRRL/mHLA-E. Four days after transduction the expression of sHLA-E molecules by LCL 721.221 cells was quantitatively verified by ELISA. Since the lentiviral construct encoding for sHLA-E contained a V5 tag verification was applied by either anti-V5 mab or anti-HLA class I (W6/32) mab as capture antibodies. An anti-b2m-HRP mab was used for detection and cell clones with the highest sHLA-E expression were used for large scale production.

### 2.4. Production of sHLA-E Molecules for the Identification of sHLA-E Peptide Ligands

For a large scale production of sHLA-E molecules sHLA-E^*∗*^01:01/LCL 721.221 cells were cultivated in bioreactors and the levels of protein production were monitored weekly. Affinity purification of sHLA-E molecules from the cell culture supernatant was performed as previously described [45]. In brief supernatants were pooled, centrifuged at 1,200 rpm for 20 min, and filtered through a 0.45 *μ*m cut-off filter (Sartorius, Göttingen, Germany). Purification was performed at pH 8.0 using NHS- (N-hydroxysuccinimide-) activated HiTrap columns, precoupled with mab W6/32. The elution of sHLA-E molecules was carried out with a 0.1 M Glycine/HCL buffer at pH 2.7.

### 2.5. Mass Spectrometric Analysis of sHLA-E Peptide Ligands

Approximately 2 mg of purified sHLA-E protein was used for peptide analysis. Purified sHLA-E molecules were treated with 0.1% trifluoroacetic acid (TFA) to elute peptides from the trimeric complexes. Separation of peptides from the hc and *β*2m was achieved by filtration through an YM membrane with a 10 kD cutoff (Millipore, Schwalbach, Germany). Subsequently the peptides were fractionated and analyzed by an Eksigent nano-LC Ultra 2D HPLC coupled to an Orbitrap ion trap mass spectrometer (Thermo Fischer, Waltham, Massachusetts, USA) resulting in a very high mass accuracy (<5 ppm). The AA sequences were assigned using MASCOT with the implemented IPI human database [46].

### 2.6. Stabilization and Stability Assay of HLA-E Surface Molecules

For stabilization of mHLA-E molecules on the cell surface of transduced T2 cells (T2E) peptide binding assays were performed. 2.5*∗*10^5^ T2E cells were incubated with range of five different concentrations with 1 *μ*M, 50 *μ*M, 100 *μ*M, 200 *μ*M, and 300 *μ*M of peptides or for cytotoxicity studies with saturated concentrations for 2.5 hours at 37°C in serum-free medium (RPMI 1640). For detection of stabilized pHLA-E complexes on the cell surface, T2E cells were incubated with the PE-labeled anti HLA-E mab 3D12 (BioLegend) for 30 minutes at 4°C. T2E cells without a peptide served as negative control. As positive control for pHLA-E surface stabilization the peptide VMAPRTLFL was carried along in the assay with a concentration of 200 *μ*M. pHLA-E surface stabilization was determined by flow cytometry (FACS Canto II, BD Biosciences, Heidelberg, Germany) and expressed as the logarithmic values of the geometric mean fluorescence intensities (gMFI) and the Fluorescence Index (FI) was calculated by the formula: FI = (log gMFI_sample_ − log gMFI_negative control_) ÷ log gMFI_negative control_. Determination of significant differences in HLA-E cell surface levels was performed by one-way ANOVA analysis, followed by Bonferroni's Multiple Comparison Test. The representative flow cytometry histograms for each peptide can be found in Supplementary Figure 1 in Supplementary Material available online at http://dx.doi.org/10.1155/2015/346714. The stability of individual p:HLA-E complexes was monitored over a time period of 6 h at 37°C and HLA-E surface levels were obtained every 2 h by flow cytometry. Decrease in HLA-E levels on the cell surface is considered as decay of p:HLA-E complexes by dissociation of the peptide from the HLA-E molecule. After peptide incubation for 2.5 h at 37°C the cells were washed two times with PBS in order to remove free peptide and subsequently resuspended in 200 *μ*L serum-free RPMI medium. For the initial time point (*t* = 0 h) HLA-E surface levels were determined immediately by flow cytometry. The cells were then further incubated at 37°C and HLA-E surface levels were determined after 2, 4, and 6 h after the initial time point.

### 2.7. Cytotoxicity Assay

The impact on NK cell mediated cytotoxicity through pHLA-E complexes was determined by a flow cytometric based cytotoxicity assay as described previously [47] with modifications. T2E cells were incubated with saturated concentrations of peptides (300 *μ*M) prior to cytotoxicity studies and used as target cells. Prior to peptide incubation the cells were labeled with 5 *μ*M CFDA SE Cell Tracer (Life Technologies, Darmstadt, Germany) for 10 minutes at room temperature. The labeled cells were washed twice in PBS and resuspended in serum-free RPMI 1640 medium. The HLA-E stabilization assay was performed for 2.5 hours at 37°C followed by coincubation with either untouched CD56^+^ NK cells freshly isolated from PBMCs by negative selection or with NKL cells with the indicated effector : target (E : T) ratios at 37°C for 4 hours. The negative selection of CD56^+^ NK cells from PBMCs was performed by the EasySep Human NK cell Enrichment Kit (Stemcell Technologies, Vancouver, Canada) according to the manufactures protocol. As a control for spontaneous cell death target cells without effector cells were incubated in serum-free medium. After the 4-hour incubation samples were incubated with 7-amino-actinomycin (7-AAD) (BioLegend, San Diego, USA) at the final concentration of 2.5 *μ*g/mL for 1*∗*10^6^ cells, and finally samples were analyzed by flow cytometry.

### 2.8. Flow Cytometric Acquisition and Data Analysis

Flow cytometric acquisition was performed using a FACS Canto II flow cytometer (BD Biosciences, Heidelberg, Germany). The stopping gate function was used to acquire 10,000 CFSE^+^ targets for each sample. For discrimination between effector and target cells the gating was done on side scatter (SSC) versus log-scale CFSE. To measure target cell death CFSE^+^ cells were gated on 7-AAD^+^ versus forward scatter (FSC) for determining the percentage of 7-AAD^+^ target cells cocultured with effector cells or spontaneous cell death of target cells in the absence of effector cells. The percentages of NK cell cytotoxicity were calculated using the following equation:
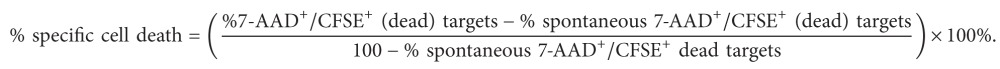
(1)


### 2.9. Modeling of pHLA-E Complexes with Noncanonical Self-Peptides

To understand the structural impact of pHLA-E complexes on NK cell function, modular models of distinct pHLA-E complexes were performed.

A limited number of HLA-E crystal structures are available, most of them containing 9-meric peptides. Since in this work self-peptides of extraordinary length were used and no structure of HLA-E^*∗*^01:01 or E^*∗*^01:03 with a long peptide is available, the HLA-E^*∗*^01:01 crystal structure 3CDG [41] with the VMAPRTLFL peptide template obtained from the protein data bank (PDB, http://www.rcsb.org/) was used for peptide mutagenesis using Coot and the implemented rotamer library to find the best side chain orientations with minimum steric clashes [48]. Each model was then subjected to energy minimization using MODELLER [49]. The graphics program PyMOL (http://www.pymol.org/) was used to generate the structure figures.

## 3. Results

### 3.1. Peptide Features

To scan the repertoire of HLA-E restricted peptides that can be acquired in the absence of HLA class I molecules we transduced the HLA class I deficient 721.221 cell line with the pRRL.PPT.SFFV.mcs.pre/sHLA-E vector encoding for the truncated version of HLA-E^*∗*^01:01 heavy chain. Following affinity purification of sHLA-E molecules, peptides were eluted and sequenced by mass spectrometry. The analysis resulted in 36 HLA-E restricted peptides, 27 of those with a length of >10 AAs (Table 1). Among those there were six 16-mer, five 15-mer, six 14-mer, five 13-mer, four 12-mer, and one 11-mer peptide(s). The source of the peptides was highly diverse, all peptides are derived from cell cycle regulatory proteins, matrix proteins, DNA repair, and stress induced proteins. Although an anchor motif could not be accurately determined, 10 out of 36 peptides were shown to contain a small hydrophobic residue (Val or Leu) at their P2 position.

### 3.2. Noncanonical Identified Self-Peptides Stabilize HLA-E Surface Expression on T2E Cells

**Figure 1:**
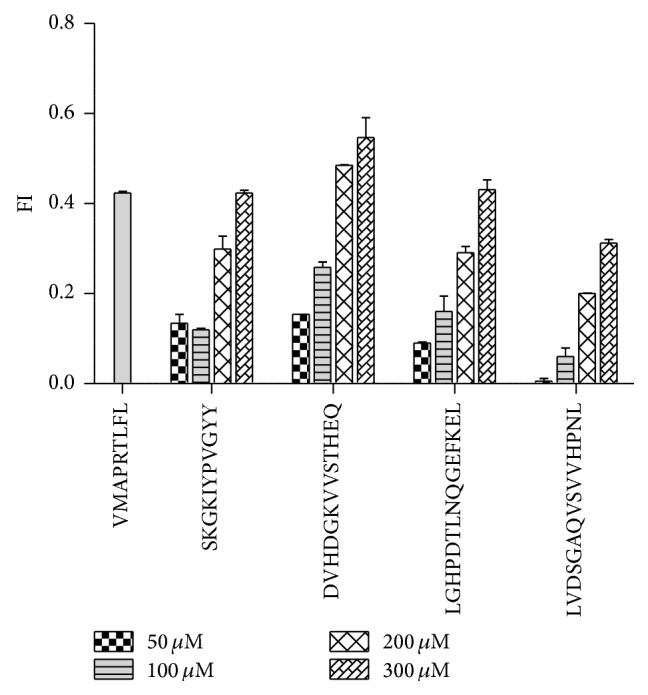
Noncanonical peptides stabilize HLA-E surface expression. The T2 cell line transduced with the HLA-E^*∗*^01:01 allele was incubated in the presence or absence of the indicated peptides at 37°C for 2.5 hours prior to staining with the mab 3D12 (anti-HLA-E) and analyzed by flow cytometry. Each peptide was titrated with the indicated concentrations and compared to a known HLA-E peptide ligand VMAPRTLFL that was carried along with a saturated concentration of 200 *μ*M indicated by the difference in median fluorescence intensity between T2E cells in the presence of peptide ± SEM of at least two individual experiments. FI values from T2E cells in the absence of peptide were subtracted from experimental samples as background control.

The capability of the sHLA-E restricted self-peptides to stabilize empty HLA-E molecules on the surface of T2E cells was tested utilizing a peptide binding assays with the four selected peptides highlighted in Table 1. The peptides were each titrated to relative saturation equilibrium of pHLA-E molecules. Incubation with the 10-mer SKGKIYPVGY and the 15-mer LGHPDTLNQGEFKEL showed a similar capacity in stabilizing HLA-E on the cell surface (Figure 1). The incubation with the 13-mer DVHDGKVVSTHEQ resulted in the highest expression of surface HLA-E molecules, whereas the 15-mer LVDSGAQVSVVHPNL peptide showed the lowest increase in HLA-E surface expression in comparison to the other peptides at the highest peptide concentration of 300 *μ*M. The capability of the four peptides to stabilize HLA-E molecules was compared to the known HLA-E peptide ligand VMAPRTLFL that is present in the leader sequence of HLA-G [28]. No significant difference in HLA-E surface expression levels could be observed between the tested peptides and the HLA-G peptide at the highest concentration tested.

### 3.3. HLA-E Restricted Self-Peptides Impact NK Cell Mediated Cytotoxicity

**Figure 2:**
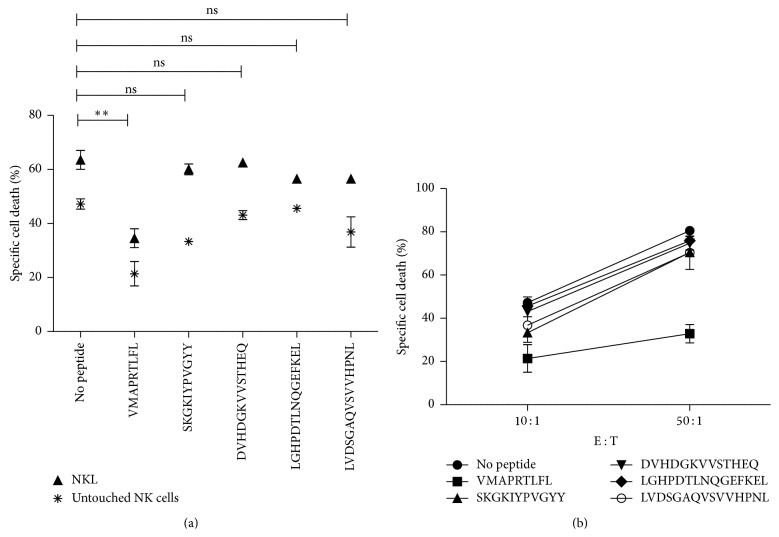
Noncanonical peptides impact on protection against NK cell cytotoxicity. (a) T2E cells were incubated in the presence or absence of peptides prior a 4-hour cytotoxicity assay with NK cells (NKL) or fresh isolated untouched CD56^+^ NK cells from PBMCs at a E : T ratio of 10 : 1 and (b) untouched NK cells at 10 : 1 or 50 : 1. Specific cell death was calculated based on the percentages of dead (7-AAD^+^) and live cells (CFSE^+^) ± SEM. Specific cell death of target cells incubated with peptide was compared to target cells without peptide incubation.

In order to determine the impact of the identified HLA-E restricted peptides on NK cell mediated cytotoxicity, we performed flow cytometry based cytotoxicity assays with peptide pulsed T2E cells. The percentage of specific lysis of target cells was determined for all four peptides as well as for target cells pulsed with the control peptide VMAPRTLFL. HLA-E^VMAPRTLFL^ complexes represent a ligand for the inhibitory NKG2A/CD94 receptor, although HLA-E^VMAPRTLFL^ was also described to bind to the NKG2C/CD94 activating receptor [53], however, with lower affinity. As expected in the absence of exogenous HLA-E peptides, T2E cells with empty HLA-E molecules on their surface were lysed by NKL and untouched NK cells, respectively (Figure 2). All four self-peptides had an impact on NK cell mediated cytotoxicity resulting in a complete loss of protection against NK cell cytotoxicity that showed no significant difference in protection with NKL or untouched NK cells compared to T2E cells without peptide. The 10-mer SKGKIYPVGYY and the 13-mer DVHDGKVVSTHEQ elicit the highest loss of protection resulting in almost equal percentages of cell death as with target cells incubated without peptide. The two 15-mer peptides LGHPDTLNQGEFKEL and LVDSGAQVSVVHPNL have a slightly lower impact on cytotoxicity but are also nonprotective HLA-E ligands compared to the VMAPRTLFL peptide that showed a significant protective effect against NK cell cytotoxicity with both NKL and untouched NK cells as it has been shown in previous reports [19, 37, 50]. This indicates that sufficient HLA-E surface levels were available for NK cell inhibition and that the difference in recognition of the other pHLA-E complexes could be due to lower stability of NKG2A/CD94 receptor, HLA-E complexes or simply no interaction at all.

### 3.4. Stabilization of HLA-E with Noncanonical Peptides Results in Stable pHLA-E Complexes

**Figure 3:**
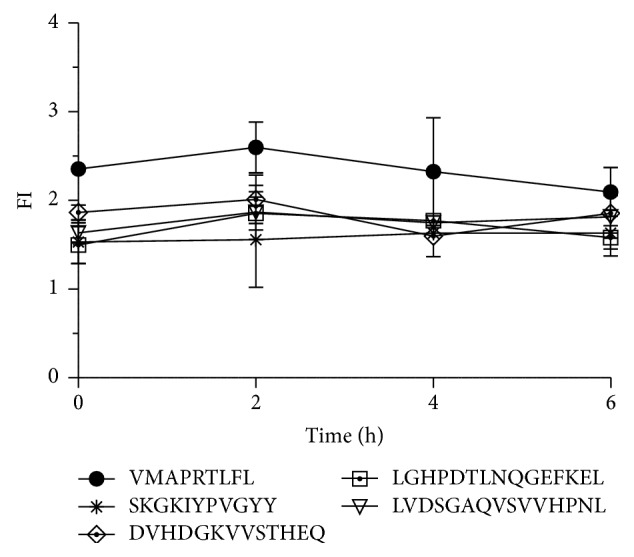
Noncanonical peptides provide stable HLA-E surface levels. T2E cells were incubated with test peptides and HLA-E surface levels were determined at four different time points with 0, 2, 4, and 6 h after the peptide stabilization assay ± SEM. The four noncanonical peptides were incubated with a concentration of 300 *μ*M, the HLA-G peptide with 200 *μ*M.

To exclude that the loss of HLA-E surface levels is the reason for the loss of cell protection against NK cell cytotoxicity we examined the stability of the pHLA-E complexes obtained by incubation with the test peptides. The HLA-E cell surface levels were determined for each tested peptide from the time point on after peptide incubation. Following the incubation with peptide T2E cells were washed to remove free peptide and further incubated at 37°C; HLA-E surface levels were determined at time points 0, 2, 4, and 6 h after peptide incubation by flow cytometry. The levels of HLA-E surface expression did not alter relatively to the initial level for all tested peptides after 6 h (Figure 3). This indicates that the pHLA-E complexes stabilized by the noncanonical test peptides provide sufficient HLA-E surface levels for the 4 h incubation during the cytotoxicity assay and additionally highlight their immunogenic potential as they provide stable HLA-E complexes.

### 3.5. Structural Modeling Reveals Extraordinary Peptide Conformations

**Figure 4:**
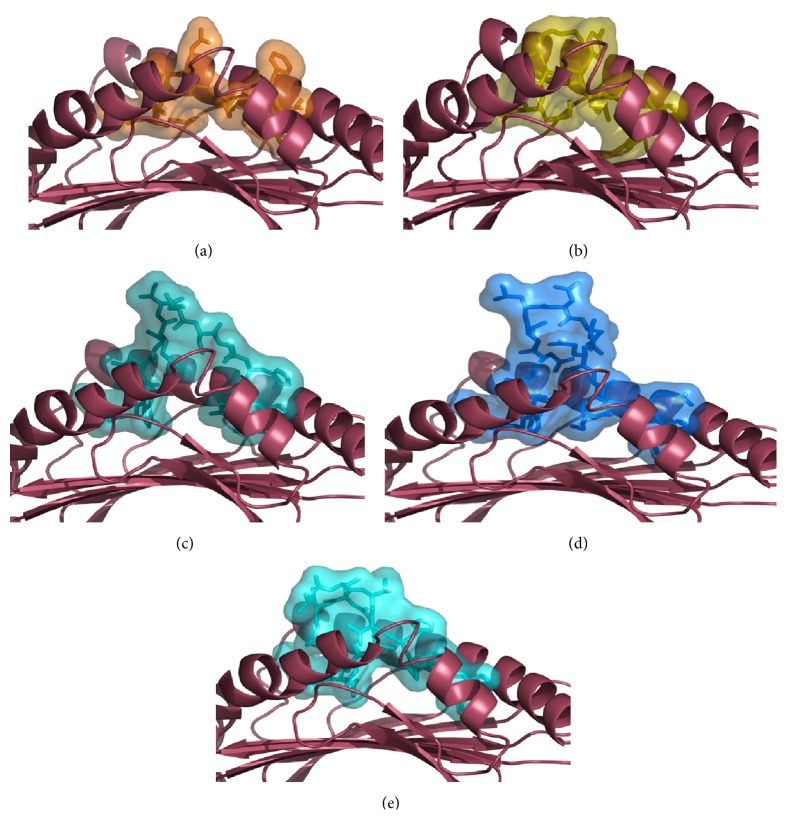
Models of noncanonical self-peptides bound to HLA-E show highly diverse confirmation resulting in distinct accessible surface areas. (a) The HLA-E crystal structure with the VMAPRTLFL (orange) peptide (PDB: 3CDG) was used as a template for modelling the peptides into the PBR of the HLA-E molecule (raspberry). (b) The SKGKIYPVGY (olive) peptide shows only minimal surface area that protrudes out the PBR compared to (c) DVHDGKVVSTHEQ (teal) that strongly forms a bulgy structure that provides large parts of its accessible surface to solvent. This is also the case for the peptides (d) LGHPDTLNQGEFKEL (marine blue) and (e) LVDSGAQVSVVHPNL (cyan).

Since no structural analysis of HLA-E bound to a peptide longer than 9 AAs is available we established a structural illustration of four selected peptides based on an available HLA-E^*∗*^01:01 crystal structure PDB ID: 3CDG [41] that shows HLA-E^VMAPRTLFL^ in complex with NKG2A/CD94. The peptides in this study were obtained from soluble HLA-E^*∗*^01:01 molecules and are highly diverse in their sequence and length. By* in silico* peptide mutagenesis of the present VMAPRTLFL peptide, we modelled the four analyzed peptides into the PBR of HLA-E^*∗*^01:01 (Figure 4). These pHLA-E complexes show striking alterations in the peptides' conformation where the 13-mer DVHDGKVVSTHEQ and the two 15-mer LGHPDTLNQGEFKEL and LVDSGAQVSVVHPNL peptides protrude outwards the PBR exposing different parts of their AA side chains to solvent. Depending on the AA composition of the peptide the accessible surface that is exposed to solvent varies significantly among the found self-peptides selected for analysis. The 10-mer SKGKIYPVGY peptide shows no significant deviance from the stretched conformation found with canonical peptides bound to HLA-E as shown with the VMAPRTLFL peptide.

## 4. Discussion

HLA-E was thought to monitor class I expression and present their signal peptides to immune effector cells; however little is known about its overall peptide repertoire. It is well established that the NK cell recognition of pHLA-E complexes is dysregulated during cellular stress or viral infections. To understand the invariability of HLA-E restricted peptides and to determine if HLA-E is able to acquire and present self-peptides other than HLA class I derived signal peptides, we utilized soluble HLA technology [42]. For the first time naturally HLA-E^*∗*^01:01 restricted peptides could be identified. These peptides that were acquired in HLA class I negative cells, mimicking a pathogenic situation, are highly diverse in regard to their features, binding motif and length with up to 16 AAs. HLA-E is known to present preferentially peptides of 8-9 AAs [52, 54, 55] in length and most of these peptides share similar features considering their overall hydrophobicity and the presence of favored AAs at primary anchor position such as a methionine at p2 or leucine at the C-terminal position of the peptide [55–57]. Available HLA-E structures are exclusively bound to nonameric peptides. The structural analysis of HLA-E^*∗*^01:01 with the peptides VMAPRTLFL, VMAPRTLLL, VMAPRTVLL, VMAPRALLL, VMAPRTLIL, and VMGPRTLIL [40, 41, 53, 57, 58] and HLA-E^*∗*^01:03 with the peptides VMAPRTVLL and VTAPRTLLL [59] revealed that positions p2, p7, and p9 are major anchor residues for peptides of 9 AAs in length and that major contacts between hydrophobic peptide residues to the HLA-E heavy chain are given by van der Waals interactions [28, 40, 54]. The sequences of identified peptides vary in their anchoring AAs and are highly divers (Table 1), even though the peptides of 9 AAs in length do not confirm the p2 or p9 anchor motif of previously defined HLA-E restricted peptides. Features of the newly identified peptides make it impossible to predict their conformation when bound to HLA-E based on the peptide binding mode represented by available pHLA-E structures. Their diversity suggests a differential binding mode within the PBR and furthermore extensive alterations of the pHLA-E landscape presented to immune effector cells. Previous studies revealed that the PBR of HLA-E is not as restricting as initially assumed and support the present findings of promiscuous epitopes.* In vitro* refolding studies demonstrated previously that HLA-E is able to bind a differential set of peptides [55] and the variability of bound peptides determine the thermal stability of pHLA-E complexes [60]. Another study by Lampen et al. identified over 500 peptides bound to HLA-E^*∗*^01:03 in TAP downregulated K562 cell lysates where these peptides showed a length up to 13 AAs and highly diverse sequence composition [61]. However, the HLA-E^*∗*^01:03 peptide repertoire identified by Lampen et al. showed no shared sequences with our findings. Six peptides that were identified from HLA-E^*∗*^01:03 are derived from shared intracellular protein sources like Calprotectin S100A9, serine/arginine matrix protein 2, Histone H2A, ubiquitin-protein ligase but differ in length and sequence compared to the peptides eluted from HLA-E^*∗*^01:01 in our data. In a recent study by our group the peptide repertoire of HLA-E^*∗*^01:03 in LCL 721.221 cell was identified [62]. HLA-E^*∗*^01:03 derived peptides revealed similarities to HLA-E^*∗*^01:01 derived peptides regarding peptide length and sequence diversity; however the pΩ position of HLA-E^*∗*^01:03 derived peptides was restricted to lysine whereas HLA-E^*∗*^01:01 peptides do not show a conserved AA at this position of the peptide. Nevertheless, the majority of HLA-E^*∗*^01:03 restricted peptides were noncanonical with a length between 11 and 16 AAs, yet no shared peptides were found among the HLA-E^*∗*^01:01 and HLA-E^*∗*^01:03 restricted peptide repertoires from the same proteome.

Based on previous findings, it could be assumed that the mode of binding of noncanonical peptides to HLA-E might be less efficient compared to HLA class I derived signal peptides which contain a sequence that enables deep binding of the peptide into the PBR of HLA-E [57]. This assumption is supported by the experimental observation in this study that high concentrations of long peptides are needed for stabilization of empty HLA-E molecules on the cell surface of T2E cells, while the concentration required when using nonameric signal peptides is less.

The four tested peptides that represent heterogenous noncanonical HLA-E ligands of the 36 peptides that were identified were selected by their differences in length and sequence for analyzing their recognition by immune effector cells. Peptides were loaded on T2E cells and used as targets for NK cells. T2E cells that were not pulsed with peptide were subsequently lysed due to the lack of protective HLA-E ligands where NK cell cytotoxicity was most likely triggered by stimulatory NK cell receptors that are expressed by NKL and polyclonal NK cells such like NKG2D or NKp46 [63, 64]. The addition of the VMAPRTLFL peptide resulted in a protective response that was expected since HLA-E^VMAPRTLFL^ was described previously to interact with the inhibitory NKG2A/CD94 receptor [65].

HLA-E molecules bound to the four selected self-peptides that differ in length and sequence showed differences in pHLA-E surface expression but provided similar HLA-E surface levels compared to the HLA-G derived peptide and furthermore stability of these surface levels throughout the monitored time course of 6 h. However, all four peptides could not restore cell protection by HLA-E resulting in high increase of target cell lysis. It is well known that HLA-E is the solely ligand for the NKG2A/CD94 receptor on NK cells [21] and peptide selective abrogation of HLA-E binding to the NKG2A/CD94 receptor has been shown by Michaëlsson et al. [30], where a HLA-E peptide ligand derived from the leader sequence of HSP60 protein provides stable HLA-E surface expression but failed in recognition by the NKG2A/CD94 receptor and were subsequently lysed by NK cells. We hypothesized that the conformational changes of the peptide that is bound to HLA-E will have a major impact on the recognition by the NKG2A/CD94 receptor.

Peptides of unusual length [4, 66, 67] and unusual conformation have been described and structurally analyzed for several HLA class I subtypes [8, 66, 68]. Commonly these long peptides form a bulgy structure whose mobility is dependent on the local structure determined by the peptide sequence. Utilizing an* in silico* peptide modelling approach (Kraemer et al., Manuscript in preparation) for a prediction of how the four long HLA-E restricted self-peptides would accumulate in the PBR shows the possible conformation of the peptides and their accessible surface to solvent (Figure 4). Depending on the AA sequence of the bound peptide, the C-terminus and N-terminus are partly buried in the PBR and start to bulge outwards the groove in the middle region of the peptide. Assuming that these predicted peptide conformations will be similar than* in vivo* it is not surprising that the complexes HLA-E^SKGKIYPVGY^, HLA-E^DVHDGKVVSTHEQ^, HLA-E^LGHPDTLNQGEFKEL^, and HLA-E^LVDSGAQVSVVHPNL^ exhibiting such diverse conformations are not recognized by the invariant receptor NKG2A/CD94. Hence, the loss or reduction of protective interaction with the NKG2A/CD94 receptor could be an explanation for the increased cytotoxicity observed when compared to the protective HLA-E^VMAPRTLFL^ complex. The structural analysis of the interaction between HLA-E^VMAPRTLFL^ and the NKG2A/CD94 receptor revealed that the peptides' positions p5, p6, and p8 play a crucial role for the recognition by the receptor. The peptides' p5-Arg guanidinium group forms a hydrogen bond with the CD94-Gln110 and the contact between p6-Thr main chain and CD94-Gln112 of the CD94 subunit is also mediated by a hydrogen bond. The p8-Phe is surrounded and contacted by the three polar CD94 residues Asn156, Asn158, and Asn160 and also interacts with Phe114. However, the NKG2A subunit of the NKG2A/CD94 receptor complex exclusively contacts the peptides' p5-Arg with residue NKG2A-Pro171 through van der Waals interactions [41]. These subtle influences of certain residues of the peptide have a major impact on the receptor recognition as it was shown with a peptide VMAPRALL derived from HLA-Cw^*∗*^07:02 where the HLA-E^VMAPRALLL^ complex could not protect from NK cell lysis compared to the HLA-E^VMAPRTLFL^ complex [37]. Given the tremendous differences in accessible surface areas of the predicted peptide conformations in our study these pHLA-E complex are most likely not exposing the AA side chains and orientation that are appropriate for the NKG2/CD94 receptor recognition. Every single peptide that is presented by a classical HLA molecule or HLA-E dictates the fade of the cell through the structural interplay between the TCR or the NK cell receptor. The structural invariability of the intrinsic HLA-E heavy chain is mediated by the sequence and structure of the bound peptide.

Taken together, HLA-E presents divers set of peptides when no HLA class I signal peptide is available. Even a difference in the peptide features between HLA-E^*∗*^01:01 and HLA-E^*∗*^01:03 [61] could be observed. In this work, a selection of four peptides that differ in length and sequence stabilized pHLA-E complexes that do not support cell protection against NK cytotoxicity, which might be based on the accessible surface of these molecules that is exposed to the NKG2/CD94 receptors. It is of note that the peptide repertoire in the HLA class I negative LCL 721.221 cell line might not be identical in other cell types when HLA class I expression is absent under clinical conditions. LCL 721.221 cells have been selected as a model to analyze the peptide specificity of HLA-E without the competition against signal peptides. However, the peptide mediated balance shift from cell protection to cell lysis was very distinct and might suggest a posttransplant role of HLA-E.

Moreover, these results emphasize the possibility that HLA-E is able to present a broad range of peptides that could underline the hypothesis of an out-competition of HLA class I in presenting mHAGs as a mechanism to reduce GvHD posttransplantation and also emphasize its role during HCMV infection.

## Supplementary Material

Histograms of HLA-E surface expression on T2E cells.
Titration of 5 distinct HLA-E peptides.

## Figures and Tables

**Table 1:** Canonical and noncanonical HLA-E peptide ligands.

Peptide sequence																Source	Reference
Canonical HLA-E peptide ligands (*reported in literature*)																	
Peptide position																	
**1**	**2**	**3**	**4**	**5**	**6**	**7**	**8**	**9**									
V	M	A	P	R	T	L	I	L								HLA-Cw signal peptide	[50]
V	M	A	P	R	T	L	F	L								HLA-G signal peptide	[24, 28]
V	M	A	P	R	T	L	L	L								HLA-A^*∗*^01 signal peptide	[51]
V	M	A	P	R	T	L	V	L								HLA-A^*∗*^02 signal peptide	[51]
A	L	A	L	V	R	M	L	I								ATP binding cassette transporter	[52]
Q	M	R	P	V	S	R	V	L								Heat shock protein 60	[30]
A	I	S	P	R	T	L	N	A								HIV gag protein	[22]
S	Q	Q	P	Y	L	Q	L	Q								Gliadin-wheat protein	[23]
S	Q	A	P	L	P	C	V	L								EBV-BZLF1 protein	[24]

Noncanonical HLA-E^*∗*^01:01 peptide ligands																	
**1**	**2**	**3**	**4**	**5**	**6**	**7**	**8**	**9**	**10**	**11**	**12**	**13**	**14**	**15**	**16**		
T	L	Q	A	S	N	Q	S	E	I	I	Q	K	Q	V	V	Isoform 2 of nucleosome-remodeling factor subunit BPTF	
I	L	D	L	S	S	M	A	P	Q	A	L	V	Q	F	W	POLQ DNA polymerase theta	
T	R	I	F	G	D	A	L	A	S	I	K	Q	Q	A	Q	Isoform 1 of serine/arginine repetitive matrix protein	
R	M	G	L	A	K	V	S	L	S	P	V	I	T	E	M	PLEC1 Isoform 6 of Plectin-1	
G	G	I	V	T	L	S	Q	A	A	G	D	V	D	A	R	Isoform 6 of Nesprin-1	
I	K	D	D	E	K	E	A	E	E	G	E	D	D	R	D	HNRPC protein	
V	P	R	L	G	S	T	F	S	L	D	T	S	M	S		CBL E3 ubiquitin-protein ligase CBL	
H	L	I	G	I	H	F	T	G	C	S	M	N	P	A		AQP6 Aquaporin-6	
V	V	T	G	N	M	G	S	N	D	K	V	G	D	F		DSG1 Desmoglein-1	
**L**	**V**	**D**	**S**	**G**	**A**	**Q**	**V**	**S**	**V**	**V**	**H**	**P**	**N**	**L**		ASPRV1 retroviral-like aspartic protease 1	
**L**	**G**	**H**	**P**	**D**	**T**	**L**	**N**	**Q**	**G**	**E**	**F**	**K**	**E**	**L**		Calprotectin S100A9	
G	R	C	I	Q	M	W	F	D	S	A	Q	G	N			FOLR3 folate receptor 3 precursor	
M	S	F	L	W	P	V	H	A	E	P	N	P	D			Putative uncharacterized protein TTC39A	
A	P	I	V	G	G	E	M	A	V	L	A	L	L			COL29A1 Isoform 1 of Collagen alpha-5(VI) chain	
G	Q	G	S	N	G	Q	G	S	S	S	H	S	S			IRS4 Insulin receptor substrate 4	
L	T	P	F	P	G	P	G	P	R	R	P	P	W			LMTK3 lemur tyrosine kinase 3	
Y	R	S	G	G	G	F	S	S	G	S	A	G	I			KRT1 Keratin, type II cytoskeletal 1	
S	L	I	N	N	H	I	P	C	L	I	S	G				Putative uncharacterized protein HMGN1	
P	K	K	T	E	S	H	H	K	A	K	G	K				Histone H2A type 3	
**D**	**V**	**H**	**D**	**G**	**K**	**V**	**V**	**S**	**T**	**H**	**E**	**Q**				KRT14 Keratin, type I cytoskeletal 14	
A	S	G	Y	V	S	S	A	D	L	V	F	T				Isoform 2 of Cytokine receptor common subunit beta	
L	D	G	K	V	I	S	F	E	G	C	A	V				Olfactory receptor 10S1	
V	S	E	K	G	T	V	Q	Q	A	D	E					7 kDa protein	
P	R	V	M	T	P	P	S	D	E	P	D					Protocadherin Fat 1	
A	V	T	F	S	S	L	P	A	A	I	T					cDNA FLJ59480, highly similar to Smoothelin	
G	P	Q	R	C	G	W	P	D	G	L	G					AP2 associated kinase 1	
P	S	L	T	S	V	T	T	T	F	V						Olfactory receptor 51D1	
**S**	**K**	**G**	**K**	**I**	**Y**	**P**	**V**	**G**	**Y**							52 kDa protein	
P	G	H	C	E	D	V	L	V	L							Isoform 2 of Urea transporter 2	
A	P	P	P	P	E	P	A	L								Homo sapiens selective LIM binding factor	
Q	P	H	P	G	D	Q	S	E								HSPA1L putative uncharacterized protein	
I	L	N	F	P	P	P	P									Isoform 2 of Caprin-2	
I	V	E	S	R	P	V	P									XRN2 Isoform 1 of 5′-3′ exoribonuclease 2	
I	I	A	I	F	G	P	G									Isoform 1 of multidrug resistance protein 3	
M	K	A	A	P	G	V	E									Solute carrier family 12, member 6 isoform c	
A	A	V	L	E	Y	L										Histone H2A type 2-B	

Canonical HLA-E peptide ligands are reported to induce HLA-E surface expression, and noncanonical HLA-E peptide ligands are peptides that were identified within this study. The peptides selected for stabilization and cytotoxicity studies are highlighted in bold.

## Abbreviations

sHLA: Soluble human leukocyte antigen
mHLA: Membrane anchored HLA
AA: Amino acid
pHLA complex: Peptide HLA complex
KIR: Killer-cell immunoglobulin-like receptor.
