# Supplementary material for: HLA-E: Presentation of a Broader Peptide Repertoire Impacts the Cellular Immune Response—Implications on HSCT Outcome
Source: Stem Cells Int. 2015 Aug 12;2015:346714. doi: 10.1155/2015/346714 (PMC4549550; doi:10.1155/2015/346714)
Supplement: Supplementary file 1 — Histograms of HLA-E surface expression on T2E cells. Titration of 5 distinct HLA-E peptides. [file 346714.f1.pdf]

Supp. Figure 1. HLA-E surface expression levels

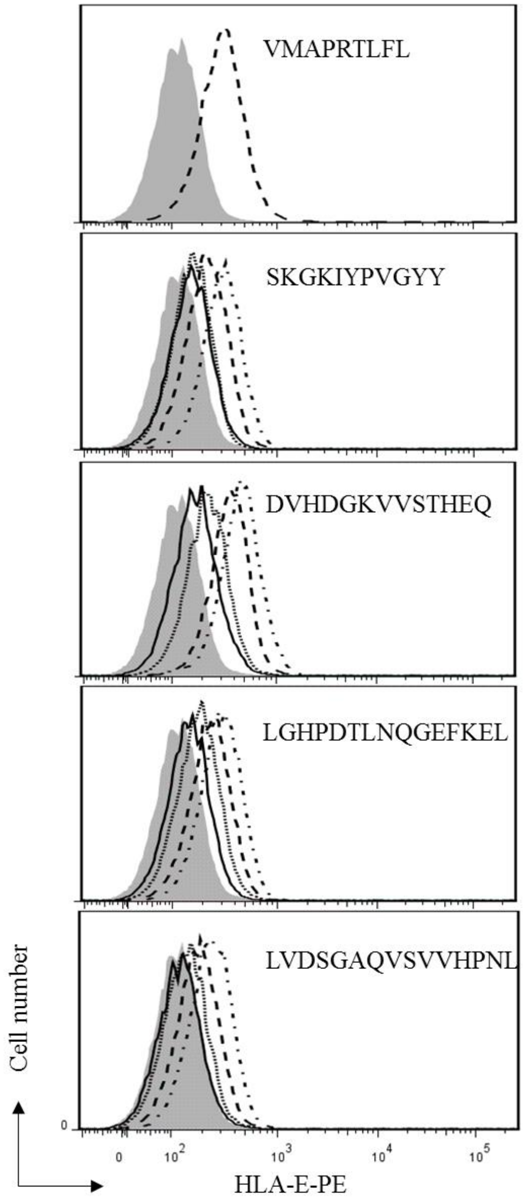

Flow cytometry histograms of the HLA-E surface expression levels on T2E cells after incubation with the test peptides. HLA-E surface levels were determined after incubation with the anti-HLA-E 3D12-PE mAb and differences in surface expression at distinct peptide concentrations are indicated by the graphs as follows: filled grey: T2E cells without peptide, solid black: 50 µM, dotted: 100 µM, dashed: 200 µM and complexed: 300 µM.
